# A Case of Circumferential Type A Aortic Dissection with Intimo–Intimal Intussusception

**DOI:** 10.3400/avd.cr.20-00104

**Published:** 2020-12-25

**Authors:** Yoichi Yamashita, Sayako Nakagawa, Shohei Kitamoto, Kosuke Sakamoto, Taiko Horii

**Affiliations:** 1 Department of Cardiovascular Surgery, Faculty of Medicine, Kagawa University, Kita-gun, Kagawa, Japan; 2 Shikoku Medical Center for Children and Adults, Zentsuji, Kagawa, Japan

**Keywords:** intussusception, aortic dissection

## Abstract

An 83-year-old woman was referred to our hospital under a diagnosis of acute aortic dissection. Contrast-enhanced computed tomography revealed no intimal flap in the mid-ascending aorta, and the intimal flap was found from the distal ascending aorta to the aortic arch. Operative findings showed that the intima of the mid-ascending aorta was circumferentially dissected and was inverted into the aortic arch. An emergent replacement of the ascending aorta was successfully performed; however, she died of a global intestinal ischemia on the fourth operative day.

## Introduction

Circumferential dissection with intimo–intimal intussusception is a rare condition in the aortic dissection. Initial report by Hufnagel and Conrad^1)^ was succeeded by less than 80 cases in type A aortic dissection. We experienced a case of circumferential type A aortic dissection with intimo–intimal intussusception.

## Case Report

**Figure 1:**
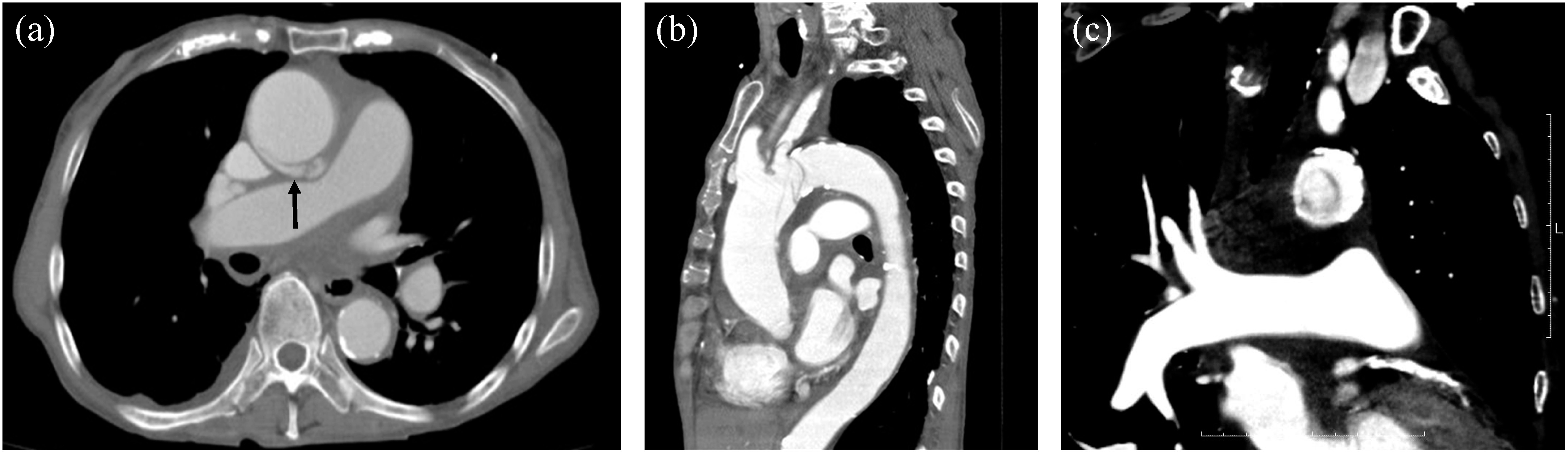
Fig. 1 Contrast-enhanced computed tomography. (**a**)–(**c**) show axial, sagittal, and oblique views, respectively. In (**a**) and (**b**), the intimal flap cannot be found in the mid-ascending aorta. (**a**) An extravasation of contrast medium to the posterior wall of the proximal ascending aorta (arrow). (**b**) An intimal flap is seen from the distal ascending aorta to the aortic arch. (**c**) An intimal flap is seen as concentric circle at the aortic arch.

**Figure 2:**
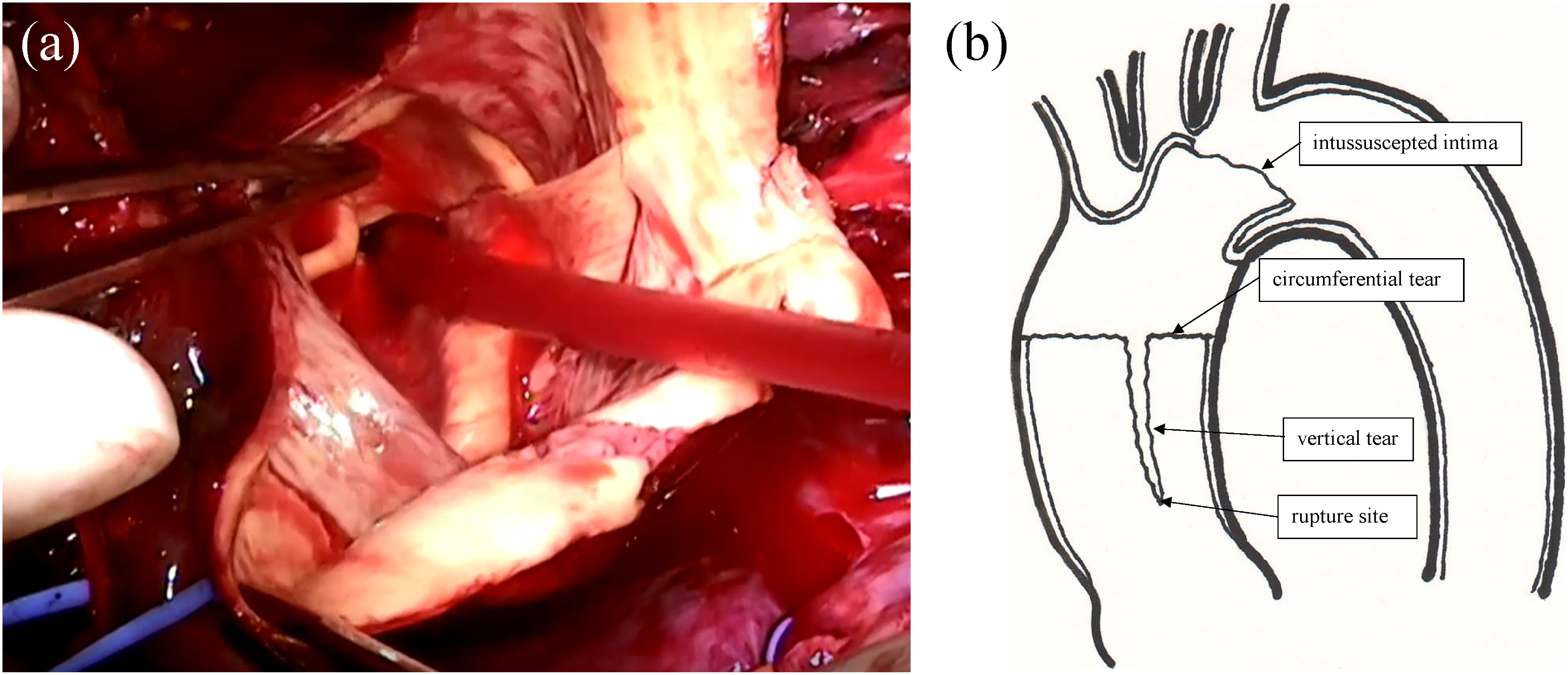
Fig. 2 The operative finding. (**a**) A suction catheter is inserted into the aortic arch through the intussuscepted intima. (**b**) A schema of the operative finding.

**Figure 3:**
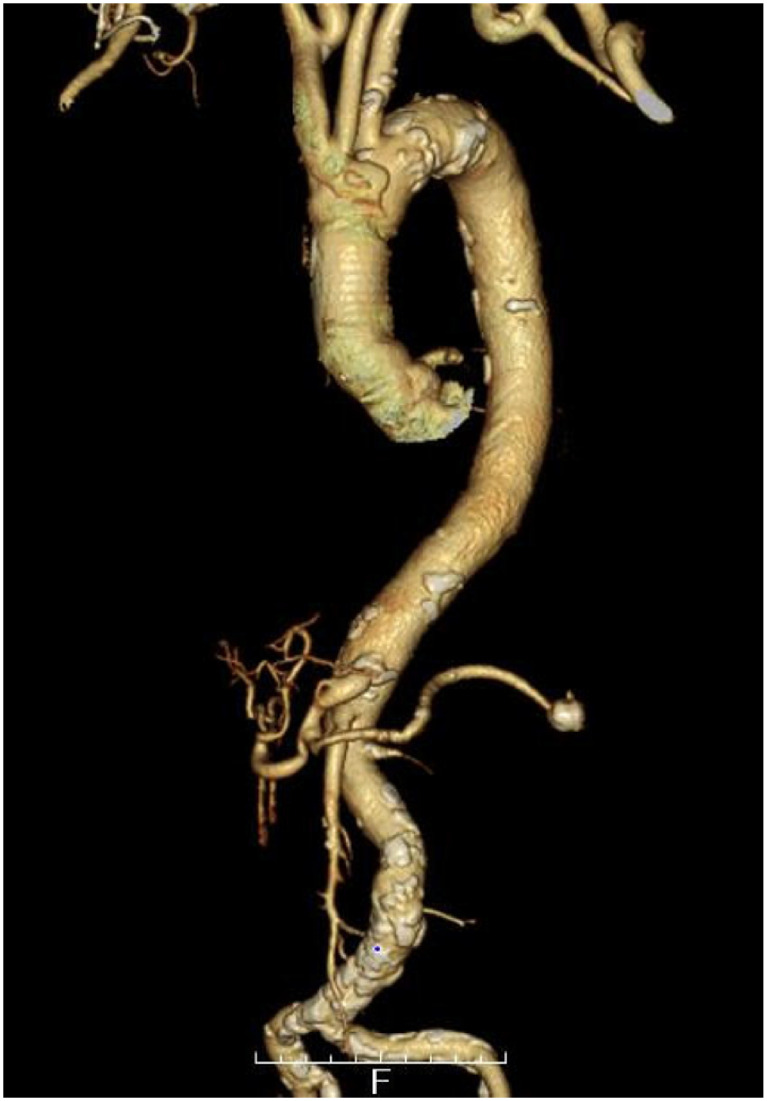
Fig. 3 Postoperative contrast-enhanced computed tomography. There is no residual dissection, and both the celiac and superior mesenteric artery are well enhanced.

An 83-year-old woman suffered from nausea and posterior headache with a history of hypertension, peripheral artery disease, and hemodialysis for 16 years. An emergent magnetic resonance imaging revealed a small infarction in the right cerebellum, and the cardiologist pointed out blood pressure difference between the right upper arm (88/55 mmHg) and the right lower leg (152/76 mmHg). Contrast-enhanced computed tomography (CT) was performed on suspicion of aortic dissection and revealed that the intimal flap was recognized in the distal ascending aorta through the aortic arch but was not found in the mid-ascending aorta (**Figs. 1a**–**1c**). She was diagnosed as type A aortic dissection with an atypical form and was transferred to our hospital for further treatment. On arrival, she was fully alert, and her hemodynamic state was stable. The CT also revealed an extravasation of contrast medium to the posterior wall of the proximal ascending aorta (**Fig. 1a**) with a slight pericardial effusion. At the emergency room, she complained of chest oppression, and her vital status rapidly deteriorated. A repeat transthoracic echocardiography revealed an increasing effusion in the pericardial cavity. She was immediately transferred to the operating room under a diagnosis of rupture of the aortic dissection. A profuse amount of pericardial effusion was bloody, but the ascending aorta was not bluish. After pericardial effusion evacuation and clot removal, excess bleeding was not found. Because the ascending aorta seemed unruptured, we started core cooling through the ascending aorta to bicaval bypass. When we divided the posterior wall of the ascending aorta, fresh bleeding came out. We hurried a cooling under compression around the posterior wall of the ascending aorta, and circulatory arrest was induced at the tympanic temperature of 25°C. A retrograde cerebral perfusion through the superior vena cava was immediately started. The ascending aorta was opened, and we found a circumferential detachment of the intima at 2 cm below the brachiocephalic artery. The intimal flap was intussuscepted distally to the aortic arch. A vertical tear reached down to 2 cm above the sino-tubular junction in the posterior wall of the ascending aorta, and the top of the vertical tear was sought to be the rupture site (**Figs. 2a** and **2b**). The inverted intima was retrieved from the aortic arch to the ascending aorta. The false lumen of the distal side extended to the arch vessel and that of the proximal edge was almost thrombosed and limited around the vertical tear. As no more tear was found distally, and the aortic valve and the coronary ostium were intact, replacement of the ascending aorta was performed using 30-mm woven polyester graft (J-Graft, Japan Lifeline, Tokyo, Japan). Reinforcement of the anastomosis site was done in the proximal and distal side with BioGlue (CryoLife Inc., Kennesaw, GA, USA) and external Teflon felt strip. The cardiopulmonary bypass was weaned uneventfully. The time of the operation, cardiopulmonary bypass, and circulatory arrest were 352, 202, and 43 min, respectively. The patient was extubated on the second operative day without any neurological deficit. On the third operative day, a metabolic acidosis progressed in spite of a continuous renal replacement therapy. The rate of water removal was 70 ml/h for 50 ml/h intake, and water balance still remained at >4,700 ml. An emergent contrast-enhanced CT revealed a global mesenteric ischemia, although no residual dissection was found and the celiac and superior mesenteric artery were patent (**Fig. 3**). Her family denied a surgical intervention as a further treatment. Although she was treated with a continuous infusion of alprostadil alfadex (0.005 µg/kg/min), she expired on the fourth operative day. Her family denied an autopsy. Histologic examination of the aortic wall taken during the operation showed cystic median necrosis.

## Discussion

Hufnagel and Conrad reported the initial case of the intimo–intimal intussusception of dissecting aneurysms in 1962,^1)^ and since then, less than 80 cases have been reported as an intimo–intimal intussusception in type A aortic dissection. Roberts et al. reviewed 35 intussusception cases in type A dissection,^2)^ and we found the other 44 such cases until April 2020. A complete or near total circumferential dissection in type A aortic dissection causes adverse complications due to the mobile cylinder-shaped flap. As classified by Sanders et al.,^3)^ these flaps intussuscept antegrade to the aortic arch^1–4)^ or retrograde to the left ventricle.^3,5–9)^ The antegrade intussusception may cause a malperfusion of the aortic arch vessels, and the retrograde intussusception causes an aortic valve insufficiency or coronary artery suffocation. Bidirectional intussusception case was also reported.^10)^

To diagnose in an acute setting, multiple modalities were used.^3,5–8,10)^ Less invasive modalities, such as an echocardiography and a CT, are frequently used.^2–10)^ An angiogram was once adopted when an angiogram was a gold standard tool.^1,2)^ Even now, when the symptom is mimicking acute coronary syndrome in the retrograde intussusception, an angiogram is selected to evaluate the disease, but it may fail to reach a correct diagnosis.^6)^ Sraow et al.^5)^ reported an echocardiography demonstrating the prolapsed intima in the retrograde intussusception. A transesophageal echocardiography (TEE) can detect the real-time motion of the intimal flap, degree of the intussusception, degree of the interference with coronary ostium and the aortic valve, and function of the left ventricle. TEE is useful to assist physicians in determining the exact diagnosis and the optimal treatment strategies.^9)^

Regarding CT, Karabulut et al.^4)^ proposed the typical finding of this phenomenon. They describe the intraluminal wind sock-like appearance filled with contrast material and surrounded by a contrast column, curvilinear intimal flap in the aortic root, absence of a flap in the dilated mid-ascending aorta, and dissection with intimo–intimal intussusception. Nowadays, multidetector-row CT and electrocardiogram-gated CT are helpful to make a clear image and understand this rare mechanism.^8)^ Although the diagnosis of aortic dissection had been made by previous hospital in our case, we could not decide the type of dissection because the intimal flap did not exist in the mid-ascending aorta as a typical aortic dissection. The increasing pericardial effusion presumably because of the rupture of the ascending aorta forced us to rush into the operating room. During the operation, we became aware of this condition, and CT findings were recognized as intussusception retrospectively. The blood pressure of the lower leg was higher than that of the right arm in our patient. The intimal flap is seen in complex fashion in sagittal view of the CT (**Fig. 1b**) but is seen in eccentric in oblique view (**Fig. 1c**). The blood flow to the descending aorta was sought to be maintained and that to the arch vessel was kept backward from the distal end of the intussuscepted intima, which may cause the decrease of the upper arm blood pressure. The cerebellum infarction of our patient was limited, and the distal portion of the arch vessels was well enhanced by the CT. Her initial symptoms might occur because of the microthrombosis that formed inside the complicatedly folded intima rather than by the result of malperfusion of the branches.

The operation comprises the retrieve and reduction of the intussuscepted intima. From the view of entry exclusion, the ascending aortic replacement is appropriate when the intimal tear exists in the ascending aorta. The aortic root reconstruction or arch procedure may be required according to the existence of another tear, degree of the aortic valve dysfunction, and suffocation of the coronary ostium or arch vessels.^4,5,7–9)^

Our patient died from mesenteric ischemia. The CT showed no residual dissection, patent celiac and superior mesenteric artery, and highly atherosclerotic change of another vessel. It was possible that a nonocclusive mesenteric ischemia occurred considering the age of the patient and long history of hemodialysis. Dehydration by continuous renal replacement therapy was done slowly; we could not prevent the ischemia. The precise cause of ischemia was unclear because autopsy was not permitted.

Making an exact diagnosis of intimo–intimal intussusception in the aortic dissection before operation is difficult. The combination of less invasive modalities, such as CT and echocardiography, helps us reach an accurate diagnosis and perform an optimal operation.

## Conclusion

We report a case of circumferential dissection with intimo–intimal intussusception in type A aortic dissection.
